# Supplementary material for: Imidacloprid Impairs Glutamatergic Synaptic Plasticity and Desensitizes Mechanosensitive, Nociceptive, and Photogenic Response of Drosophila melanogaster by Mediating Oxidative Stress, Which Could Be Rescued by Osthole
Source: Int J Mol Sci. 2022 Sep 5;23(17):10181. doi: 10.3390/ijms231710181 (PMC9456553; doi:10.3390/ijms231710181)
Supplement: Supplementary file 1 [file ijms-23-10181-s001.zip › ijms-1861034-supplementary.pdf]

Imidacloprid impairs glutamatergic synaptic plasticity and de-sensitizes mechanosensitive, nociceptive, and photogenic re-sponse of *Drosophila melanogaster* by mediating oxidative stress, which could be rescued by osthole

Chuan-Hsiu Liu [1,2,a], Mei-Ying Chen [3,a], Jack Cheng [3,4], Tsai-Ni Chuang [3], Hsin-Ping Liu [3,*] and Wei-Yong Lin [3,4,5*]

[1] Graduate Institute of Chinese Medicine, China Medical University, Taichung 40402, Taiwan
[2] School of Chinese Medicine, China Medical University, Taichung 40402, Taiwan
[3] Graduate Institute of Integrated Medicine, China Medical University, Taichung 40402, Taiwan
[4] Department of Medical Research, China Medical University Hospital, Taichung 40402, Taiwan
[5] Graduate Institute of Acupuncture Science, China Medical University, Taichung 40402, Taiwan
[a] C.-H. Liu and M.-Y. Chen contributed equally to this work.
* Correspondence: hpliu@mail.cmu.edu.tw (HPL) & linwy@mail.cmu.edu.tw (WYL)

Supplementary Table S1. Primers used in this study.

Gene Name	5' primer	3' primer
norpA	TCTTTGAGCCTGTCACGTTG	TCTTTGCTCTTGCCCTTGAT
ninaC	GCACAGCCAGTTTGTGAAGA	GCCTTGGTCAGTTGATTGGT
trp-F:	GATTACGGCATTACCGAGGA	CAACTCCCTGCGACTTCTTC
trpl	GAACAGCGGAATGGATGTTT	TGGACTCCACCTTGATCTCC
Trpγ	TGTGGTGCTGCTCAACCTAC	GTTTGGAGCGAGCAAACTTC
Itpr-F	TTGCGGTTCCTTCAGCTACT	CGTCTCCGATACCAGGTTGT
rdgC	TCTGCTGGACACGGATGTTA	CAATCGCAGTTTCGAACTCA
inaC	ATTTCATGGGATCGTTCTCG	AATGTTCACCCTCCACTTGC
inaD	GCAGACCACCAACAACAATG	GTGGTATTCCGCTTCTGCAT
inaE	ATTCCACGGACTTCGATGAG	TGCCTTTACGGTTCGATCTC
Gprk1	GCCAACATACTGCTCGATGA	CATGAGTTCCCACTGAAGCA
Galphaq	AGGACAAGCGTGGGTACATC	CTCGAACGTGGTAACGGTCT
Gβ76C	TATCGCTGGCTTATCGCTTT	TCGTGACCGAAGAACATCTG
Gγ30A	CGCTGGCCGTTATCTAAATC	GGCCCATGGATTGTTCTTC
Pkc53E	AAGTGCAACCGCCATTTAAG	GTGGGCGTCAAGTCTGTTTT
Cam	GACACCGATAGCGAAGAGGA	GTCATCACGTGACGCAACTC
nompc	AACTCTGCGGGTGTTCAAGT	ACTTAGGAGCAAACCGACCA
nan	CCGAGCGGTTGAAGAACTTT	CTGCACCTCCTTCATCTCCT
TRPA1	CGGCATCTATGTGGTCATGT	AAGGACAAGTGGTTCGGTTG
pain	TTTCCTGCTCTTCGTGATCTT	TCCGCCTGAGCCTTAATAACT
Arr2	GGAGTGATTGTGGTGGAACC	CGACCATAGCGATAGGTGGT
nrv2	AAGACGCAGCCAGAAAAGC	GCAGGTAGTTGACAGCACCA
TotA	CCAAAATGAATTCTTCAACTGCT	GAATAGCCCATGCATAGAGGA
TotC	AATGAATGCCTCCATTTCTCTACTA	CTCGTCAGAATAGCCCAAGC
TotM	CAAGCCTGCACTATGAATCCT	CATCTTCGTTCTCAGCATTTACC
TotX	CCTCAAGGAGGATCATTTGG	AGACGAAATATACCGGGTTCC
TRPM	GATTCTGGAGGGACTGATCG	GAAGGCTTCGAAGGTGACAG
GstD1	TCGCGAGTTTCACAACAGAA	TGAGCAGCTTCTTGTTCAGC
GstD2	CGGACATTGCCATCCTGT	TGCTGAAGTCGAACTCACTAACTT
GstE1	CATGTCGAGCTCTGGAATTGT	GTTTGACGGTCCTCACACAG
Sod1	GTCGACGAGAATCGTCACCT	GGAGTCGGTGATGTTGACCT
Sod3	TCAGCATGGGTGCTCACTAT	TAATGCCCGTGGAGTTGG
Cat	TGACTACAAAAACTCCCAAACG	TTGATTCCAATGGGTGCTC
Ho	GCCAATGCCTGCCCAGGTTA	ACGCGCCTTAGTTTGGCCTT
Irc	GCCCAGCTGCCTTAACTATG	GCTGCGGTGAAACCATAGAG

Supplementary Table S1 (continued). Primers used in this study.

Gene Name	5' primer	3' primer
Keap1	CAAGGAGTCGGAGATGTCG	GTAGAGGATGCGTGACATGG
Cnc	TTAACCAGACGGATGGCTTT	TGTCAACCATGCGACAGAAG
Fdh	CAGTGGTGTTGGGACATGAG	GGGGATGTAGAGAGCGATGA
Cyp6w1	AAAAACCTCTTCTTTGCACGA	TGTCCTGCAAGTTCTTTCCA
Cyp6a2	GCGCAACGAGATCCAAAC	GGGCACCAGTGTGTAGAGC
Cyp6a8	CAAGATAAGGTTCGGGCTGA	CAATGGTGTACAGTCGCAGAGT
Cyp6g1	GATGTACACCCTTATGCAGGAGA	TTCTTCTCTCCACGCCTCTG
Cyp12d1-d	GGTCCCGTTCGATCTTCAA	GGTCTTGTGCTCCTCCGTTA
Cyp18a1	TCCACCATTCTGGAGTCGAT	ACCCATTGAGTTCCACATCC
Mdr65	TCAGCTGCCATTCCAAGATA	GATGACCCGCGATCAGAG
Jafrac1	CCCACCAAGTCCAAGGAGTA	TTCAGCGAACAGATAGCCTCT
